# Convergent validity, responsiveness, and meaningful within-subject change of the PROM-Ataxia in spinocerebellar ataxias

**DOI:** 10.1007/s00415-026-14108-1

**Published:** 2026-09-09

**Authors:** Kristofoor E. Leeuwenberg, Teije H. van Prooije, Colette Reniers, Kirsten C. J. Kapteijns, Roderick P. P. W. M. Maas, Bart P. van de Warrenburg

**Affiliations:** Department of Neurology, Donders Institute for Brain, Cognition, and Behaviour, Radboud University Medical Center, Geert Grooteplein Zuid 10, 6525 GA Nijmegen, Netherlands

**Keywords:** Spinocerebellar ataxia, Patient-reported outcome measure, Trial readiness, Validity

## Abstract

**Background:**

The clinimetric properties of the Patient-Reported Outcome Measure of Ataxia (PROM-Ataxia) have only been partially explored. This study aimed to investigate its convergent validity, responsiveness, and minimal clinically important difference (MCID), as well as its discriminative ability at the earliest disease stages in different types of spinocerebellar ataxia (SCA).

**Methods:**

Baseline and 1 year PROM-Ataxia data were obtained from three single-center cohort studies involving ataxic and pre-ataxic SCA1, SCA3, and SCA7 mutation carriers and healthy controls. Spearman correlations were assessed between PROM-Ataxia scores and the Scale for the Assessment and Rating of Ataxia, Inventory of Non-Ataxia Signs, SCA Functional Index, Cerebellar Cognitive Affective Syndrome Scale, 5-level EuroQoL 5-Dimensional Visual Analogue Scale, Patient Health Questionnaire−9, Unified Huntington Disease Rating Scale Part IV, and the Activities of Daily Living subscale of the Friedreich Ataxia Rating Scale. Responsiveness was evaluated using standardized response means (SRM), and MCIDs through anchor-based and distribution-based approaches.

**Results:**

Seventy-six mutation carriers (20 SCA1, 40 SCA3, 16 SCA7) were included at baseline, with 68 completing the 1-year follow-up visit. PROM-Ataxia scores correlated moderately to strongly with other measures (ρ=0.46–0.89, *p*<0.001) and differentiated pre-ataxic individuals from healthy controls (*p*<0.001). Over one year, PROM-Ataxia demonstrated a gradual average increase (+ 3.9, *p*=0.048) with considerable between-subject variability of change (SD=22.0) and low responsiveness (SRM=0.17). The MCID of the PROM-Ataxia total score was estimated at 9.8–12.7.

**Conclusion:**

PROM-Ataxia already distinguishes pre-ataxic SCA mutation carriers from healthy controls in physical, ADL, and mental health domains, and demonstrates strong convergent validity. Although responsiveness *at the group level* was somewhat limited because of large interindividual variability, 1 year follow-up scores adequately reflected the *individual* patient’s global impression of change.

**Supplementary Information:**

The online version contains supplementary material available at https://doi.org/10.1007/s00415-026-14108-1.

## Introduction

Spinocerebellar ataxias (SCAs) constitute a heterogeneous group of rare inherited conditions characterized by gradually progressive degeneration of the cerebellum. Although ataxia is the hallmark symptom shared by all SCAs, its rate of progression differs and various extra-cerebellar symptoms and signs may emerge depending on the specific subtype [1]. Furthermore, in polyglutamine (polyQ) SCAs, the size of the CAG expansion affects the clinical phenotype, with larger expansions typically causing more severe, earlier-onset disease [2]. Together, this results in a large phenotypic heterogeneity.

To date, there are no disease-modifying treatments available for SCAs. However, several promising therapeutic developments are currently in progress such as nucleotide-based gene silencing, especially in the case of polyQ SCAs [3]. Given this development, there is an urgent need for appropriate outcome measures to monitor changes in the disease burden of SCA over time, ideally within the (brief) timeframe of a clinical trial. Over the past years, the Scale for the Assessment and Rating of Ataxia (SARA) has been the instrument of choice in clinical studies because it is easily applicable and extensively validated. However, the SARA is based only on clinical scoring of ataxia, does not incorporate any non-ataxia symptom or the patient’s experience, and the clinical meaningfulness of some of its items may be questionable [4, 5]. Therefore, additional tools are warranted to capture the full extent of the disease burden in this heterogeneous population.

To address this need, the Patient-Reported Outcome Measure of Ataxia (PROM-Ataxia) was recently developed through a multi-phase process involving the active engagement of ataxia patients [6]. The aim of this new instrument was to measure the comprehensive disease burden from the patient's perspective, complementing existing clinician-rated assessments and biomarker measures. It comprises a 3-domain, 70-item questionnaire covering motor functions, psychosocial and cognitive aspects, and activities of daily living (ADL). The initial publication indicated that it is a valid and reliable instrument, with the anticipation that it will also prove responsive over time [6].

Recent studies of PROM-Ataxia in American and Chinese SCA populations have demonstrated a high internal consistency and strong correlation with other ataxia measures [7–9]. Furthermore, one of these studies suggested that the scale may be comparable or even more sensitive to changes over time than current measures like the SARA and other, non-ataxia-specific PROMs [7]. However, longitudinal studies in large populations still remain limited and, as highlighted in the original development paper, validation across diverse cohorts, patient groups, and cultures is important to obtain a better understanding of the clinimetric properties and utility of PROM-Ataxia as a potential outcome measure. Importantly, its sensitivity to detect abnormalities at the earliest disease stages – in pre-ataxic individuals – has not been explored so far.

We here examined PROM-Ataxia in a single-center, combined Dutch cohort of SCA1, SCA3, and SCA7 mutation carriers over a one-year period. Specifically, we aimed to evaluate its (i) discriminative ability at the earliest disease stages, (ii) convergent validity, (iii) responsiveness over time, and (iv) meaningful within-patient change. With regard to the latter, we used different, tailored anchors to examine minimal clinically important differences (MCID) of the PROM-Ataxia total score, as well as its individual domain subscores.

## Methods

### Study design and participants

Baseline and 1-year follow-up data were collected from three distinct observational cohort studies on SCA types 1, 3, and 7, all conducted at Radboud university medical center. Participants were included based on a molecularly confirmed diagnosis, an age of 18 years or older, and the absence of any other neurological disorders. Disease stages were categorized according to Klockgether et al. [10]: stage 0 = no gait difficulties, stage 1 = onset of gait difficulties, stage 2 = loss of independent gait, and stage 3 = confined to a wheelchair. In addition, based on their SARA score, participants were classified as pre-ataxic (SARA < 3) or ataxic (SARA ≥ 3) carriers [11]. Predicted time to or from disease onset was determined for all participants using models by Tezenas du Montcel et al. [12] for SCA1 and SCA3 and Van de Warrenburg et al. [13] for SCA7. Healthy adults without a medical history of neurological or psychiatric disorders from the SCA3 study were included as a control group to evaluate the discriminative ability of the instrument at the earliest disease stages. Since PROM-Ataxia was introduced at the first follow-up visit in the SCA1 cohort, this visit was considered the new baseline for those participants. All three studies were approved by the Medical Research Ethics Committee (MREC) Oost-Nederland, and written informed consent was provided by each participant.

### Clinical assessments

PROM-Ataxia is a 70-item patient-reported questionnaire designed to assess the impact of cerebellar disease across three domains [6]. The physical domain evaluates motor symptoms such as gait, limb coordination, and oculomotor control. In line with previous work, we aggregated both sections (one capturing symptom frequency and the other symptom severity) in our analysis because of their overlapping content [7, 8]. The activities of daily living (ADL) domain measures the impact of the disease on everyday tasks including household chores, self-care, and work. Finally, the mental domain is divided into two sections: Sect. 1 focuses on neuropsychological symptoms such as mood, anxiety, and social interaction, while Sect. 2 assesses cognitive complaints. Each item is rated on a 5-point scale, yielding a total score between 0 (no impairment) and 280 points (maximum impairment). The short form comprises a selection of 10 key items derived from the different domains of the full version.

Alongside PROM-Ataxia, eight clinical assessments—encompassing clinician-rated, performance, and patient-reported outcome measures—were selected based on their overlap with the construct of PROM-Ataxia. These included the SARA [14], Inventory of Non-Ataxia Signs (INAS) [15], SCA Functional Index (SCAFI) [16], Cerebellar Cognitive Affective Syndrome Scale (CCAS-S) [17], 5-level EuroQol 5-Dimension Visual Analogue Scale (EQ-5D-5L VAS) [18], Patient Health Questionnaire−9 (PHQ-9) [19], Unified Huntington’s Disease Rating Scale Part IV (UHDRS-IV) [20], and the Activities of Daily Living subscale of the Friedreich Ataxia Rating Scale (FARS-ADL) [21]. SARA and INAS are clinician-rated scales assessing severity of ataxia (range: 0–40) and non-ataxia signs (range: 0–16), respectively. SCAFI measures functional performance through three tasks: timed 8-m walk (8 MW), manual dexterity with the 9-hole peg test (9HPT), and articulation speed via the number of PATA repetitions in 10 s. The CCAS-S represents a cognitive screening tool specifically designed for patients with cerebellar dysfunction, providing both a raw score (range: 0–120) and a fail count (range: 0–10). Patient-reported measures included the EQ-5D-5L VAS for health-related quality of life (range: 0–100), the PHQ-9 for depressive symptoms (range: 0–27), and the FARS-ADL for daily functioning (range: 0–36). Finally, the UHDRS-IV is a clinician-rated questionnaire evaluating functional aspects of the patient’s daily life. For the PROM-Ataxia, CCAS-S, EQ-5D-5L VAS, PHQ-9, UHDRS-IV, and FARS-ADL, validated Dutch translations were used. Not all outcomes were available for every participant: the INAS and UHDRS-IV were not included in the SCA3 study protocol, and the CCAS-S and FARS-ADL were only partially completed in the SCA1 cohort due to their later incorporation into the protocol. An overview of the available clinical assessments per cohort is shown in Table 1.

**Table 1:** Baseline demographic and clinical characteristics

Demographics					
	SCA1	SCA3	SCA7	Combined	Healthy controls
Number of participants	20	40	16	76	16
Age (years)	54.7 ± 14.1	54.2 ± 10.1	49.9 ± 17.6	53.4 ± 13.0	56.5 ± 9.1
Sex (male/female)	11/9	20/20	5/11	36/40	9/7
Repeat length	44.1 ± 4.6	66.6 ± 2.8	39.6 ± 8.7	−	−
Predicted disease duration	11.1 ± 8.6	10.7 ± 9.8	5.2 ± 16.3	9.6 ± 11.3	−
Pre-ataxic (SARA < 3)	3 (15.0%)	10 (25.0%)	4 (25.0%)	17 (22.4%)	−
Klockgether stages					
Stage 0: normal	4 (20.0%)	5 (12.5%)	3 (18.8%)	12 (15.8%)	16 (100%)
Stage 1: onset walking difficulties	9 (45.0%)	16 (40.0%)	6 (36.5%)	31 (40.8%)	−
Stage 2: walking support required	4 (20.0%)	16 (40.0%)	4 (25.0%)	24 (31.6%)	−
Stage 3: wheelchair-bound	3 (15.0%)	3 (7.5%)	3 (18.8%)	9 (11.8%)	−
Clinical assessments					
SARA	12.1 ± 8.4	10.6 ± 6.9	12.5 ± 10.2	11.4 ± 8.0	0.7 ± 0.7
INAS	6.4 ± 2.7	−	4.1 ± 2.7	5.4 ± 2.9 (N = 36)	−
8 MW	6.3 ± 1.7 (N = 17)	6.0 ± 1.7 (N = 36)	6.1 ± 2.7 (N = 11)	6.1 ± 1.9 (N = 64)	4.5 ± 0.6
9HPT	42.8 ± 36.7	33.5 ± 13.6	45.0 ± 55.8 (N = 15)	40.3 ± 33.2 (N = 75)	20.3 ± 2.2
PATA	20.1 ± 5.3	27.0 ± 7.3	19.8 ± 7.2	23.7 ± 7.6	31.3 ± 5.0
CCAS-S – raw score	90.0 ± 5.6 (N = 5)	86.6 ± 14.6	87.3 ± 17.9	87.1 ± 14.9 (N = 61)	95.4 ± 10.0
CCAS-S—failures	2.0 ± 1.2 (N = 5)	2.9 ± 2.4	2.7 ± 2.5	2.8 ± 2.4 (N = 61)	1.8 ± 1.4
EQ-5D-5L VAS	69.6 ± 13.7	74.2 ± 17.3	70.0 ± 18.0	72.1 ± 16.5	93.3 ± 8.5
PHQ9	5.2 ± 3.7	5.2 ± 4.9	4.6 ± 3.2	5.1 ± 4.3	0.9 ± 1.3
UHDRS-IV	21.2 ± 5.6	-	16.6 ± 9.2 (N = 9)	19.8 ± 7.1 (N = 29)	−
FARS-ADL	5.4 ± 6.2 (N = 5)	11.8 ± 6.7	8.2 ± 8.4	10.3 ± 7.4 (N = 61)	0.13 ± 0.34
PROM-Ataxia (total)	80.3 ± 53.6	87.5 ± 53.9	83.9 ± 54.5	84.8 ± 53.3	10.4 ± 7.1
PROM-Ataxia physical	46.8 ± 28.7	54.5 ± 29.1	47.5 ± 27.6	51.0 ± 28.5	5.3 ± 3.4
PROM-Ataxia ADL	16.4 ± 17.2	16.4 ± 17.1	20.0 ± 23.9	17.2 ± 18.6	0.1 ± 0.3
PROM-Ataxia mental (1)	9.2 ± 7.0	10.2 ± 8.1	9.6 ± 7.1	9.8 ± 7.5	3.4 ± 3.4
PROM-Ataxia mental (2)	7.9 ± 6.4	6.4 ± 5.3	6.8 ± 3.9	6.9 ± 5.4	1.7 ± 2.0
PROM-Ataxia short form	14.7 ± 11.7	16.0 ± 10.5	16.3 ± 11.8	15.7 ± 11.0	0.4 ± 0.8

Data represent mean ± standard deviation or frequency (percentage)

*SCA* Spinocerebellar ataxia, *SARA* Scale for the assessment and rating of ataxia, *INAS* Inventory of non-ataxia signs, *8 MW* 8-m walking time, *9HPT* 9-hole peg test, *CCAS-S* Cerebellar cognitive affective syndrome scale, *EQ-5D-5L VAS 5-level* EuroQol 5-dimension visual analogue scale, *PHQ-9* Patient health questionnaire−9, *UHDRS-IV* Unified huntington’s disease rating scale part iv, *FARS-ADL* Friedreich ataxia rating scale—activities of daily living subscale, *PROM-Ataxia* Patient-reported outcome measure of ataxia, *ADL* Activities of daily living

Reasons for missing data:

8 MW (SCA1, SCA3, SCA7): unable to walk independently or walking aid unavailable; one participant with severe vertigo

9HPT (SCA7): one participant with severe vision loss

FARS-ADL & CCAS-S (SCA1): outcome measures were introduced later in the study protocol

INAS & UHDRS-IV (SCA3): outcome measures not included in the study protocol

The Patient Global Impression of Change (PGIC) scale was used during the 1 year follow-up visit in the SCA3 cohort as an anchoring method to determine the MCID of the PROM-Ataxia, as well as its individual domains. Using a 7-point Likert scale (1 = very much improved, 2 = much improved, 3 = minimally improved, 4 = no change, 5 = minimally worse, 6 = much worse, 7 = very much worse), participants were asked in an interview with the investigator whether they had perceived changes over the last year in gait, ADL, mental health, cognitive abilities, and quality of life. Self-reported changes in these measures were used as anchors for PROM-Ataxia’s physical domain, ADL domain, mental Sect. 1, mental Sect. 2, and total score, respectively.

### Statistical analysis

Baseline demographic and clinical characteristics of the four groups were compared using one-way analysis of variance (continuous variables) and Chi-square tests (categorical variables). Spearman correlations between PROM-Ataxia’s individual domain scores and other patient-reported, clinician-reported, and performance outcome measures were employed to assess convergent validity. Associations between longitudinal changes in PROM-Ataxia scores and these other measures were explored in a similar way.

Wilcoxon signed-rank tests were used to assess the ability of PROM-Ataxia to distinguish healthy controls from pre-ataxic SCA mutation carriers at baseline and to examine changes in parameters between baseline and 1-year follow-up with effect sizes (r) computed from the Wilcoxon Z-statistics. To further investigate determinants of PROM-Ataxia changes over time, multivariable linear regression analyses were conducted with baseline SARA score, age, sex and SCA subtype as the independent variables. Responsiveness was evaluated based on the standardized response mean (SRM), which is defined as the mean change divided by the standard deviation of change.

Within the SCA3 cohort, MCIDs of the PROM-Ataxia were estimated using two methods [22]. First, within the anchor-based approach, the aforementioned PGIC categories tailored to each individual PROM-Ataxia domain as well as its total score were utilized. Minimal clinically important differences were derived from mean PROM-Ataxia (sub)score changes in the categories ‘minimally worse’ and ‘minimally improved’. Second, the distribution-based approach defined MCID as half the standard deviation of change, and was used as a reference. All statistical analyses were performed using R Studio version 2024.04. The significance level was set at 0.05.

## Results

### Baseline demographic and clinical characteristics

A total of 76 SCA mutation carriers, including 20 with SCA1, 40 with SCA3, and 16 with SCA7, participated in this study. In addition, data from 16 healthy controls were included. Demographic and clinical characteristics of the different genotypes at baseline are outlined in Table 1. Age and sex distribution were comparable across the study groups. Relative to the other SCA groups and healthy controls, the SCA7 cohort had a slightly younger mean age (49.9 years versus 54.2–56.5 years), a shorter predicted disease duration (5.2 years versus 10.7–11.1 years), and higher proportion of women (69% versus 44–50%), though none of these differences were statistically significant (all *p* values > 0.05). The four disease stages, as defined by Klockgether, were represented within each of the three SCA cohorts. In the combined cohort, 15.8% of participants were in stage 0 (no walking difficulties), 40.8% in stage 1 (onset of gait difficulties), 31.6% in stage 2 (walking support required), and 11.8% in stage 3 (wheelchair-bound).

### Discriminative ability

PROM-Ataxia scores varied widely across participants, with an average of 84.8 and a standard deviation of 53.3 points in the combined cohort. The different SCA genotypes showed comparable average scores: 80.3 (SD 53.6) for SCA1, 87.5 (SD 53.9) for SCA3, and 83.9 (SD 54.5) for SCA7. The scale was able to clearly differentiate pre-ataxic SCA mutation carriers (i.e., SARA score < 3) from healthy controls, with significant differences observed in the total score (*p* < 0.001, r = 0.58), physical domain subscore (*p* < 0.001, r = 0.62), ADL domain subscore (*p* = 0.036, r = 0.26), mental Sect. 2 subscore (*p* = 0.018, r = 0.41), and short form score (*p* < 0.001, r = 0.68). Only the mental Sect. 1 did not show a statistically significant difference (*p* = 0.088, r = 0.30). Mean PROM-Ataxia scores for healthy controls and pre-ataxic individuals were 10.4 (SD 7.1) vs. 34.1 (SD 28.0) for the total score; 5.3 (SD 3.4) vs. 20.0 (SD 14.5) for the physical domain; 0.1 (SD 0.3) vs. 2.7 (SD 6.9) for the ADL domain; 3.4 (SD 3.4) vs. 7.2 (SD 7.4) for mental Sect. 1; 1.7 (SD 2.0) vs. 4.2 (SD 3.4) for mental Sect. 2; and 0.4 (SD 0.8) vs. 4.6 (SD 4.8) for the short form. Furthermore, PROM-Ataxia scores reflected a clear worsening from disease stage 0 to 3 (Supplementary Fig. 1).

### Convergent validity

**Fig. 1:**
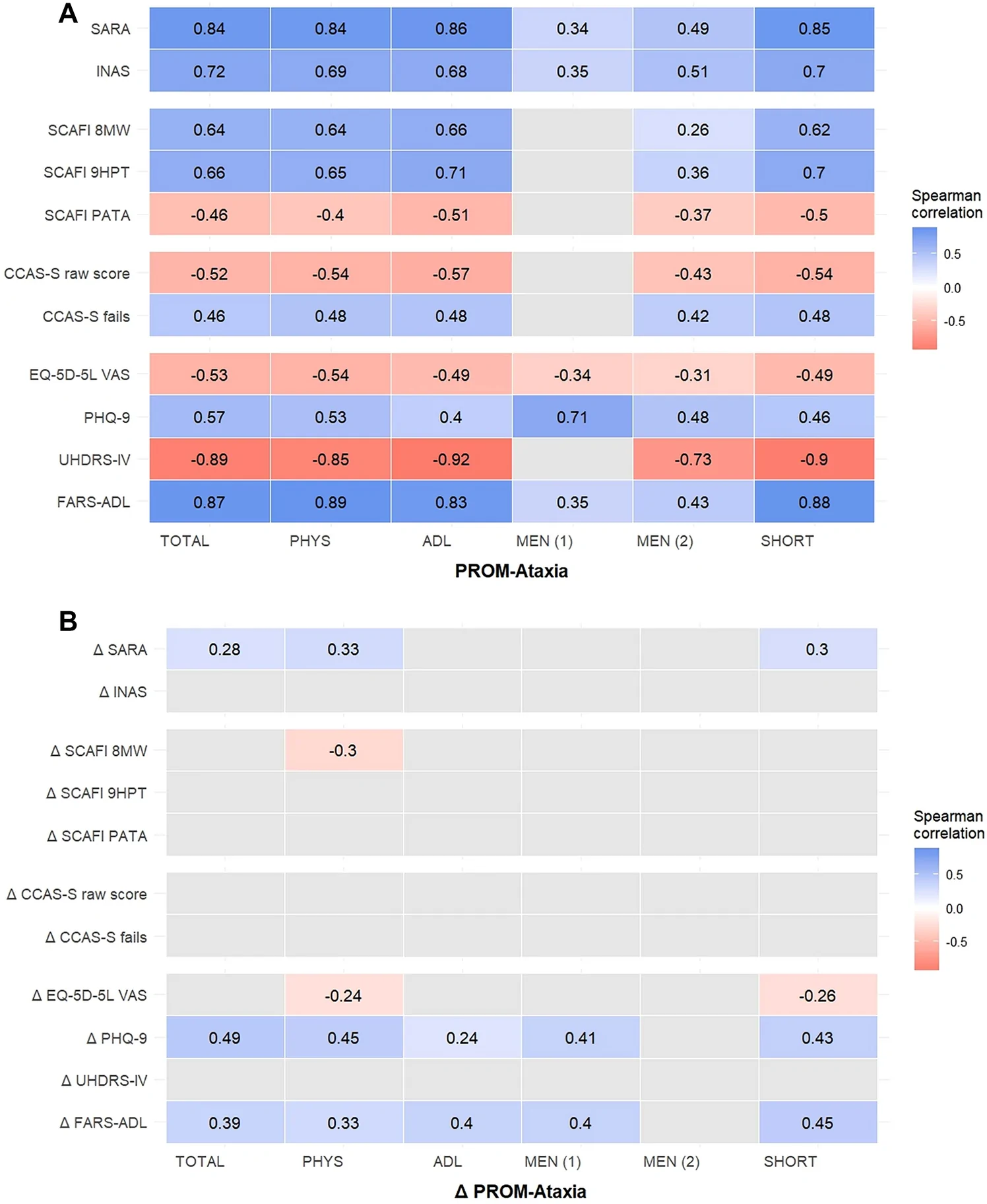
Convergent validity. **A:** Spearman correlation heatmap demonstrating convergent validity of the PROM-Ataxia with other outcome measures at baseline. Only correlations with a *p* value < 0.05 are shown; non-significant correlations are represented by grey boxes. **B:** Correlations between longitudinal changes in PROM-Ataxia scores and other outcome measures over a 1‑year period. *TOTAL* total score, *PHYS* physical domain, *ADL* activities of daily living domain, *MEN (1)* mental domain Sect. 1, *MEN 2* mental domain Sect. 2, *SHORT* short form

Associations between PROM-Ataxia scores and other clinical outcomes with overlapping constructs are shown in Fig. 1. Analyses revealed moderate to strong correlations between PROM-Ataxia total score and other disease measures (ρ = 0.46–0.89, all *p* values < 0.001). Particularly strong correlations were seen with the SARA, UHDRS-IV, and FARS-ADL (ρ = 0.84–0.89, all *p* values < 0.001), reflecting that PROM-Ataxia scores increase alongside (i) worsening clinical ataxia severity and (ii) greater disability reported by both clinicians and patients. Comparably strong correlations were found for the PROM-Ataxia physical (ρ = 0.40–0.89, all *p* values < 0.001) and ADL domains (ρ = 0.40–0.92, all *p* values < 0.001), as well as the PROM-Ataxia short form (ρ = 0.46–0.90, all *p* values < 0.001). Furthermore, the mental Sect. 1 score, representing psychosocial well-being, showed a notable correlation with the PHQ-9 (ρ = 0.71, *p* < 0.001). Over one year, correlations between changes in PROM-Ataxia and other outcome measures were weaker, although significant associations remained for the SARA, PHQ-9, and FARS-ADL (ρ = 0.28–0.49, *p* < 0.001–0.021).

### Longitudinal changes and responsiveness

**Fig. 2:**
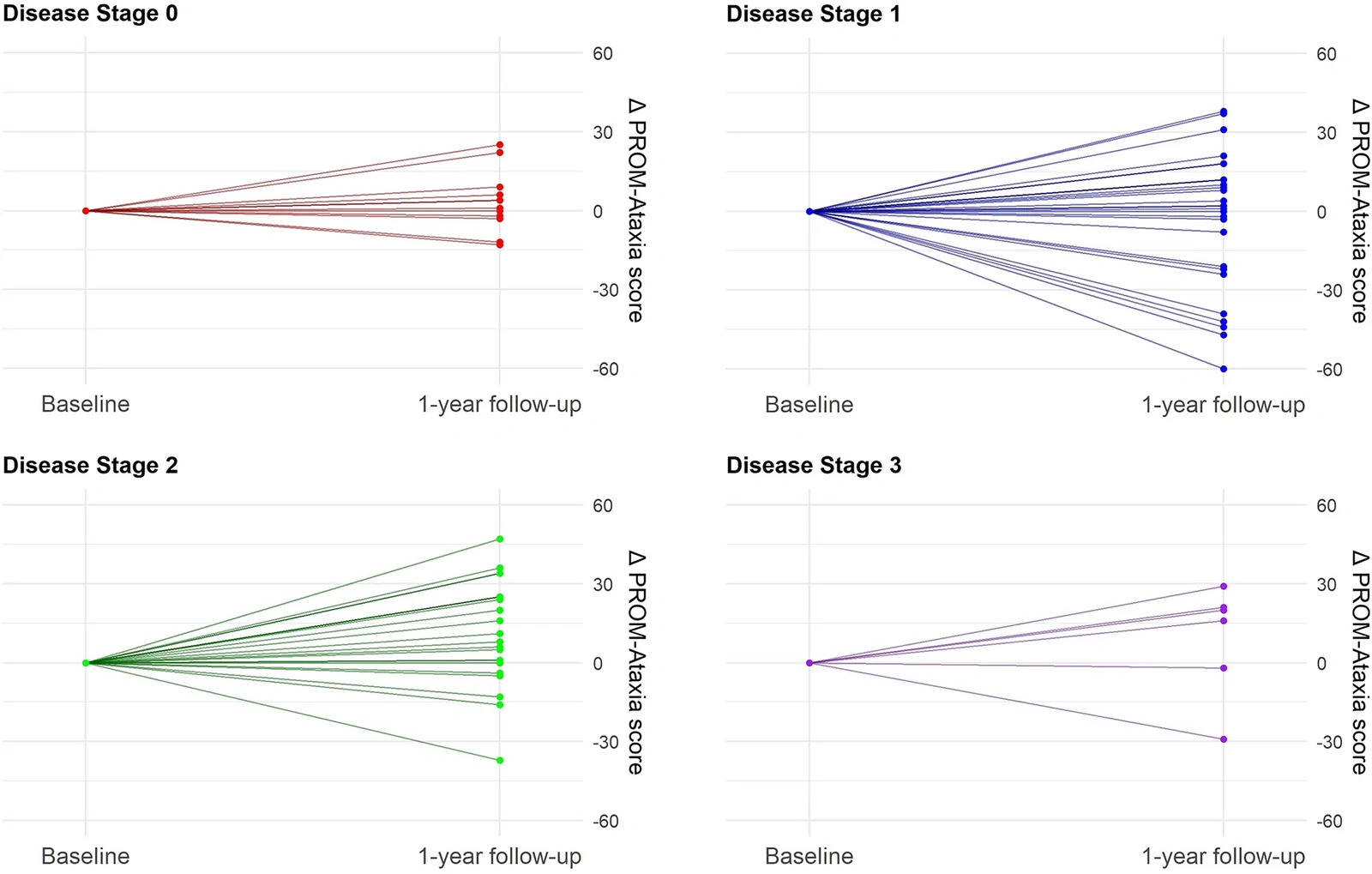
PROM-Ataxia within-subject change. Spaghetti plots showing individual 1 year PROM-Ataxia score changes in SCA mutation carriers, stratified by Klockgether disease stage (0–3). Baseline values are aligned to a common starting point to highlight individual trajectories over time

Eight SCA mutation carriers (3 SCA1, 2 SCA3, 3 SCA7) were unable to complete the one-year follow-up visit, resulting in 68 participants with longitudinal data. In the combined SCA cohort, the SARA score increased on average with 1.6 points (*p* < 0.001), FARS-ADL score with 1.4 points (*p* < 0.001), and PROM-Ataxia total score with 3.9 points (*p* = 0.048) (Table 2). Of the PROM-Ataxia domains, only ADL showed a significant increase of 1.7 points (*p* = 0.006). One‑year changes in PROM‑Ataxia scores were characterized by substantial standard deviations: 22.0 for the total score, 13.6 for the physical domain subscore, 7.9 for ADL subscore, 4.2 for mental Sect. 1 subscore, 2.8 for mental Sect. 2 subscore, and 4.9 for the short form score. Because of the large standard deviation, PROM-Ataxia demonstrated a low SRM value of 0.17, indicative of very low responsiveness at the group level. This was considerably lower than the SRM values observed for SARA (0.82) and FARS-ADL (0.47). The variability in PROM-Ataxia scores over time was observed at all disease severity stages (Table 2, Fig. 2).

**Table 2:** Longitudinal changes over 1 year

	Combined N = 68	Stage 0 N = 12	Stage 1 N = 28	Stage 2 N = 22	Stage 3 N = 6	Healthy controls
Δ PROM-Ataxia (total)	+3.9 ± 22.0* SRM: 0.17	+3.4 ± 11.5 SRM: 0.30	−2.8 ± 25.8 SRM: − 0.11	+11.0 ± 19.9* SRM: 0.55	+9.2 ± 21.3 SRM: 0.43	−1.3 ± 4.5 SRM: − 0.29
- Δ PROM-Ataxia physical	+1.2 ± 13.6 SRM: 0.09	+2.1 ± 6.5 SRM: 0.32	−1.2 ± 17.3 SRM: − 0.07	+3.5 ± 12.1 SRM: 0.29	+2.3 ± 10.3 SRM: 0.23	−0.7 ± 2.9 SRM: − 0.24
- Δ PROM-Ataxia ADL	+1.7 ± 7.9* SRM: 0.21	+0.5 ± 1.3 SRM: 0.38	−1.2 ± 7.2 SRM: − 0.17	+6.1 ± 8.8* SRM: 0.70	+1.2 ± 10.0 SRM: 0.12	0.0 ± 0.4 SRM: 0
- Δ PROM-Ataxia mental (1)	+0.6 ± 4.2 SRM: 0.13	+0.8 ± 3.5 SRM: 0.22	+0.1 ± 4.3 SRM 0.02	+0.3 ± 4.4 SRM: 0.06	+3.3 ± 4.3 SRM: 0.78	−0.5 ± 2.4 SRM: − 0.21
- Δ PROM-Ataxia mental (2)	+0.4 ± 2.8 SRM: 0.13	+0.1 ± 1.9 SRM: 0.04	−0.5 ± 3.1 SRM: − 0.15	+1.1 ± 2.6 SRM: 0.43	+2.3 ± 2.9 SRM: 0.81	−0.1 ± 1.2 SRM: − 0.08
- Δ PROM-Ataxia short form	+0.1 ± 4.9 SRM: 0.03	−0.2 ± 2.4 SRM − 0.07	−1.2 ± 5.5 SRM: − 0.22	+1.5 ± 5.1 SRM: 0.29	+2.2 ± 3.8 SRM: 0.58	−0.1 ± 0.7 SRM: − 0.14
Δ SARA	+1.6 ± 2.0** SRM: 0.82	+1.0 ± 1.1* SRM: 0.89	+1.7 ± 2.1** SRM: 0.82	+1.8 ± 2.1* SRM: 0.86	+1.6 ± 2.4 SRM: 0.76	+0.4 ± 1.3 SRM: 0.31
Δ FARS-ADL	+1.4 ± 2.8** SRM: 0.47	+0.1 ± 1.4 SRM: 0.08	+1.1 ± 3.3 SRM: 0.30	+1.7 ± 2.3* SRM: 0.73	+4.2 ± 3.3 SRM: 1.1	+0.2 ± 0.4 SRM: 0.5

Data represent mean ± standard deviation

Wilcoxon signed-rank test, * *p* < 0.05; ** p < 0.001; SRM = standardized response mean

Overview of changes in clinical assessments over the 1 year follow-up stratified according to Klockgether’s staging system, including standardized response mean (SRM) calculations as an indicator of responsiveness

### Predictors of change in PROM-Ataxia score

Using multivariable linear regression analysis, we explored potential determinants of PROM‑Ataxia change, defining total score change as the dependent variable. Baseline SARA score, age, sex, and SCA subtype were included as possible predictor variables, but none of them were significantly associated. In the SCA3 cohort, however, when PGIC quality of life was added to the model, it emerged as a significant predictor, with worsening linked to an increase in PROM‑Ataxia score (B = 13.6, *p* = 0.031). Likewise, a comparable model revealed PGIC quality of life to be a determinant of annual change in PROM-Ataxia short form score in the SCA3 group (B = 2.8, *p* = 0.035).

### Meaningful within-subject change

MCID estimations were calculated for PROM‑Ataxia total score and for each of its subscores in the SCA3 cohort (Table 3). Spearman correlations between the various PROM-Ataxia domains and their corresponding PGIC anchors revealed weak to moderate associations (ρ = 0.22–0.49), which were significant (*p* < 0.05) for all domains except for the PROM-Ataxia total score (*p* = 0.180) and short form (*p* = 0.066). The anchor-based MCID for the PROM‑Ataxia total score was estimated at 9.8–12.7 points per year. For the physical domain, which was linked to PGIC-gait, the MCID was 3.8 points per year. Corresponding values were 3.2 points per year for the ADL domain, 1.8–2.9 points per year for mental Sect. 1, 1.8 points per year for mental Sect. 2, and 1.6–3.7 points per year for the short form. When applying the distribution-based method, defined as one-half of the standard deviation of change, the resulting values were 11.0 points per year for the total score, 6.8 points per year for the physical domain, 4.0 points per year for the ADL domain, 2.1 points per year for mental Sect. 1, 1.4 points per year for mental Sect. 2, and 2.5 points per year for the short form. An overview of all MCID estimates is given in Supplementary Table 1.

**Table 3:**
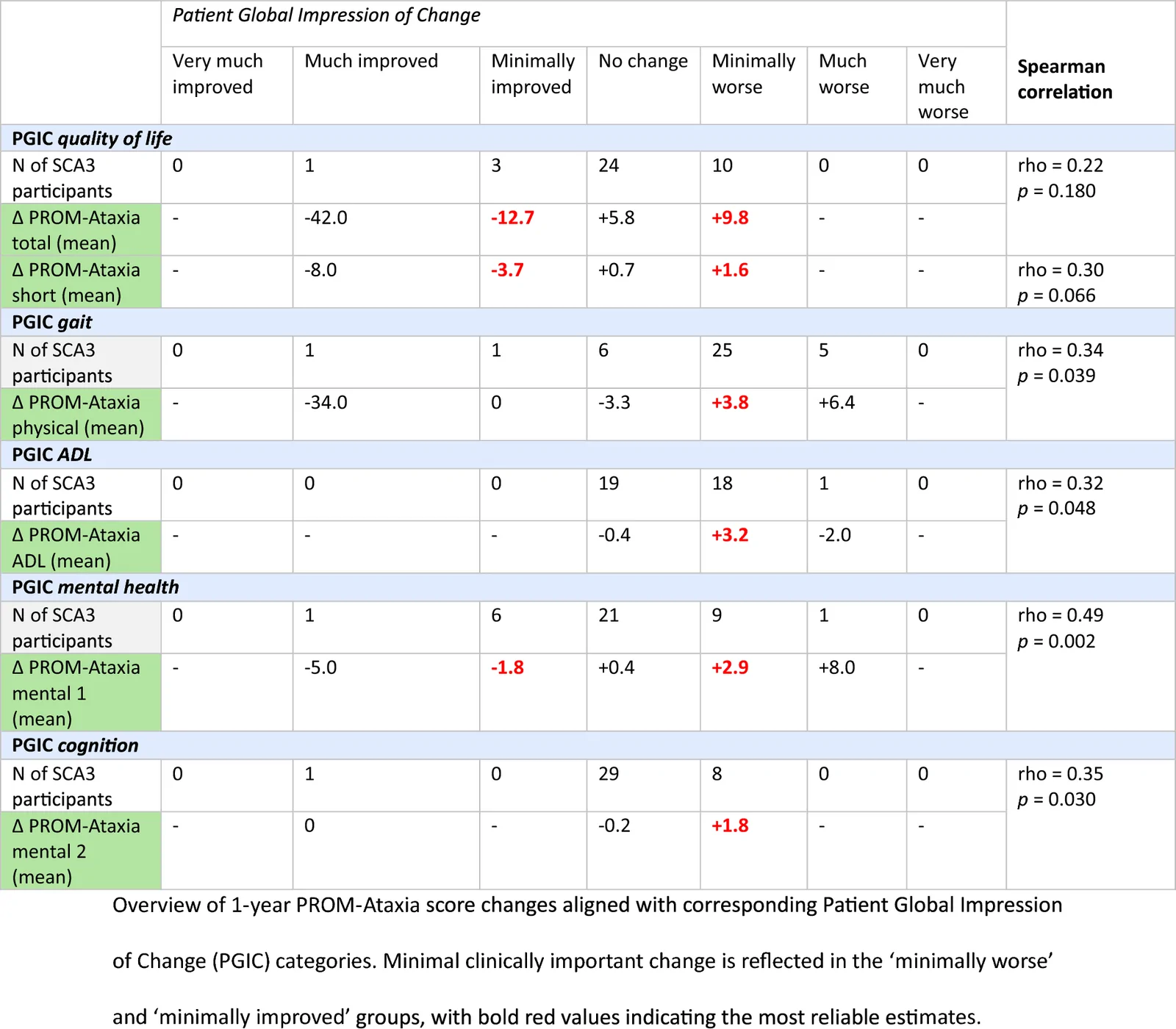
Anchor‑based assessment of the minimal clinically important difference (MCID)

Overview of 1 year PROM‑Ataxia score changes aligned with corresponding Patient Global Impression of Change (PGIC) categories. Minimal clinically important change is reflected in the ‘minimally worse’ and ‘minimally improved’ groups, with bold red values indicating the most reliable estimates

## Discussion

In this pooled analysis of three polyQ SCA cohorts, we evaluated various clinimetric properties of the PROM-Ataxia. The main findings of this study are as follows. First, PROM-Ataxia demonstrated strong associations with a diversity of clinically relevant ataxia measures, supporting its convergent validity to capture the overall disease burden. Second, by including a control group of healthy adults, we showed that PROM-Ataxia is capable of detecting early abnormalities in physical, ADL, and mental health domains in pre-ataxic individuals. Third, responsiveness of the instrument *at the group level* was lower than that of SARA and FARS-ADL, mainly because of considerable interindividual variability in 1-year changes. Fourth, by integrating anchor-based approaches in the SCA3 cohort, our results nonetheless highlight that 1-year follow-up scores adequately reflect the *individual* patient’s global impression of change. Finally, we provide estimates for meaningful within-subject changes of PROM-Ataxia total score and domain subscores.

Our data revealed strong associations between PROM-Ataxia and clinical rating scales, performance tests, and both clinician‑ and patient‑reported outcomes, providing confirmation of its construct validity. The associations with PROM-Ataxia total score observed here were comparable to those reported in recent studies, such as for the FARS-ADL (ρ = 0.87 versus 0.69–0.95), and were slightly higher for SARA (ρ = 0.84 versus 0.44–0.83) [7–9]. Moreover, our findings provide valuable insight into the construct validity of the scale’s different domains, demonstrating their capacity to capture distinct disease characteristics. This is for example illustrated by the strong link between the PHQ-9 and the PROM-Ataxia mental Sect. 1, which focuses on psychosocial symptoms. Finally, correlations with the 10‑item short form were nearly identical to those of the complete scale, which underscores the representativeness of this abbreviated version [6]. If PROM‑Ataxia is used as a secondary outcome measure, the short form may be considered from a feasibility perspective.

Besides the anticipated increase in PROM-Ataxia score as a function of ataxia severity, an important finding of our study was that the instrument already distinguished pre-ataxic SCA mutation carriers from healthy controls. This observation confirms its discriminative capacity at the earliest disease stages and suggests that pre-ataxic individuals may already experience symptoms that impact on domains captured by PROM-Ataxia. Of note, previous studies with non-ataxia-specific PROMs, such as those evaluating health-related quality of life, depression, and sleep quality, have shown that it is difficult to detect differences between pre-ataxic individuals and healthy controls, which clearly highlights an added value of PROM-Ataxia [23–25].

Over the course of one year, PROM‑Ataxia revealed a gradual, statistically significant progression at the group level consistent with the degenerative nature of these diseases. The pattern showed a degree of non‑linearity, with changes tending to be larger in more advanced disease stages (Table 2). Yet, considerable between‑subject variability was observed, resulting in a level of responsiveness that was lower than that reported in a prior longitudinal study by Burt et al. [7]. Our findings are more in line with those recently published by Rawlings et al., which also showed substantial variability and which yielded mostly non‑significant changes after 12 months [8]. Although variability in our study was consistent across all disease stages and unrelated to baseline ataxia severity or demographic factors, it did not appear to be entirely random. Indeed, PGIC quality of life was identified as a significant predictor of PROM-Ataxia change in the SCA3 cohort, suggesting that patients’ perceived well-being may partly drive the observed variability, with subjective improvement linked to lower PROM-Ataxia scores over time. An illustrative example is the largest outlier in the cohort, who showed a 60-point improvement after adopting a new balance-focused physical therapy method despite (an objective) one point progression on the SARA score. This indicates that PROM-Ataxia captures more than the disease construct of cerebellar ataxia alone, reflecting broader aspects of patients’ lives. Although this can offer interesting information regarding subjective disease burden, capturing these external influences increases variability which may be undesirable when using PROM-Ataxia as an outcome measure in a trial with only one to two years of follow-up.

Similar to PROM-Ataxia, both SARA and FARS-ADL scores increased significantly after one year of follow-up. SRMs of the latter were considerably higher than that of PROM-Ataxia, however, and appeared to follow distinct patterns during the disease course. Responsiveness of SARA was consistently high across disease stages, while responsiveness of FARS-ADL tended to be low initially with an exponential increase after the loss of independent gait. Notably, the SARA score demonstrated a relatively high SRM compared with previous SCA studies, although reported values vary widely (0.82 in our study versus 0.20–0.98) [7, 26]. These findings support the notion that the PROM‑Ataxia may best be applied as a complementary tool alongside currently adopted measures, including the more objective SARA score.

In the SCA3 cohort, we examined the MCID of PROM‑Ataxia for each component of the scale, using domain‑specific PGIC scales as anchors. Accurate interpretation requires a robust link between the anchor and the outcome measure [22]. Significant correlations were found for all domains and corresponding anchors, except for the total and short form scores. However, since the anchor linked to both scores demonstrated significance in multivariable regression, the relationship was still considered robust enough for interpretation. Of note, our anchor-based MCID values were very comparable to those recently reported by Rawlings and colleagues (Supplementary Table 1) [8]. Additional analyses using a distribution‑based method importantly yielded largely comparable outcomes. Together, the consistency of findings across both approaches as well as across both studies supports the robustness of the results.

Our study has several limitations. First, missing data were present across several of the outcome measures, mainly because the three cohort studies did not have fully identical protocols or, in some cases, introduced certain clinical assessments only at a later stage. Second, the sample size for the anchor-based MCID estimations was restricted to the SCA3 cohort and therefore somewhat limited. Nonetheless, as also mentioned earlier, the obtained values were very similar to those of a mixed polyQ ataxia cohort from the United States. Finally, our study population was limited to three SCA subtypes (SCA1, SCA3, and SCA7) and included a relatively large proportion of pre‑ataxic participants (17 out of 76), which should be taken into account when considering external validation to a broader ataxia population.

In the context of a clinical trial, PROM-Ataxia may serve as a valuable outcome assessment, complementing clinical and other measures such as SARA with a thorough patient-reported perspective. Our study confirms the construct validity and provides strong support that the instrument can serve this role effectively, even already in pre-ataxic individuals. However, a number of aspects need to be carefully considered upon its application in a trial setting. The notable variability in change over time may necessitate large sample sizes to demonstrate effects strictly related to the disease construct—an aspect that may pose a challenge in rare ataxias. Furthermore, the observed variability seems to be partly driven by external factors beyond the disease construct in a stricter sense, which may be considered either informative or problematic depending on the specific purpose and design of the trial. In specific settings, it may be useful to take into account factors that modify the overall impact of the disease; on the other hand, for evaluation of treatment effects in a drug trial, it is likely undesirable to have substantial influence from such external factors. Based on these findings, PROM-Ataxia results are best interpreted together with (i) more objective assessments of disease severity, (ii) self-perceived global impression of change, and (iii) systematic documentation of patient-relevant contextual factors.

## Supplementary Information

Below is the link to the electronic supplementary material.

Supplementary file2 (DOCX 43 KB)

Supplementary file3 (DOCX 36 KB)

## Data Availability

Data will be made available from the corresponding author upon reasonable request from any qualified investigator.

## Abbreviations

ADL: Activities of daily living
CCAS-S: Cerebellar cognitive affective syndrome scale
EQ-5D-5L VAS: 5-Level euroqol 5-dimension visual analogue scale
FARS-ADL: Friedreich ataxia rating scale-activities of daily living subscale
INAS: Inventory of non-ataxia signs
MCID: Minimal clinically important difference
MREC: Medical research ethics committee
PGIC: Patient global impression of change
PHQ-9: Patient health questionnaire−9
PolyQ: Polyglutamine
PROM: Patient-reported outcome measure
PROM-Ataxia: Patient-reported outcome measure of ataxia
SARA: Scale for the assessment and rating of ataxia
SCA: Spinocerebellar ataxia
SCAFI: SCA functional index
SD: Standard deviation
SRM: Standardized response mean
UHDRS-IV: Unified Huntington’s disease rating scale part IV
8 MW: 8-Meter walking time
9HPT: 9-Hole peg test
