# Supplementary material for: Convergent validity, responsiveness, and meaningful within-subject change of the PROM-Ataxia in spinocerebellar ataxias
Source: J Neurol. 2026 Sep 9;273(10):583. doi: 10.1007/s00415-026-14108-1 (PMC13558426; doi:10.1007/s00415-026-14108-1)
Supplement: Supplementary file 1 [file 415_2026_14108_MOESM1_ESM.docx]

**Supplementary Figure 1**. **Boxplots of baseline PROM-Ataxia scores per Klockgether disease stage.**
