# Supplementary material for: Convergent validity, responsiveness, and meaningful within-subject change of the PROM-Ataxia in spinocerebellar ataxias
Source: J Neurol. 2026 Sep 9;273(10):583. doi: 10.1007/s00415-026-14108-1 (PMC13558426; doi:10.1007/s00415-026-14108-1)
Supplement: Supplementary file 2 [file 415_2026_14108_MOESM2_ESM.docx]

**Supplementary Table 1.** Overview of the estimated minimal clinically important changes, derived via the distribution-based approach (half of the standard deviation of 1‑year change) in the full SCA cohort and the anchor-based approach based on patient global impression of change in our SCA3 cohort, compared to results previously reported by Rawlings et al.^1^

|  | **Distribution-based method**  **(Combined cohort: SCA1, 3 and 7)** | **Anchor-based method  (SCA3 cohort)** | **Rawlings et al [1]** |
| --- | --- | --- | --- |
| ∆ PROM-Ataxia total | 11.0 | 9.8 - 12.7 | 8 |
| ∆ PROM-Ataxia physical | 6.8 | 3.8 | 3 |
| ∆ PROM-Ataxia ADL | 4.0 | 3.2 | 2 |
| ∆ PROM-Ataxia mental 1 | 2.1 | 1.8 - 2.9 | - |
| ∆ PROM-Ataxia mental 2 | 1.4 | 1.8 | - |
| ∆ PROM-Ataxia short form | 2.5 | 1.6 – 3.7 | - |

[1] Rawlings AM, Chuang RS, Schmahmann JD, et al (2025) Longitudinal Changes in Patient- and Clinical-Reported Outcomes in Early Spinocerebellar Ataxia Types 1, 2, 3, and 6 from the IDEA Study. Mov Disord Clin Pract 12(5): 638-647. doi:10.1002/mdc3.14323
